# Editorial: Epigenetic biomarkers for cancer risk stratification and patient management

**DOI:** 10.3389/fgene.2024.1421500

**Published:** 2024-05-08

**Authors:** Miljana Tanić, Simone Ecker, Iben Lyskjær

**Affiliations:** ^1^ Department of Experimental Oncology, Institute for Oncology and Radiology of Serbia, Belgrade, Serbia; ^2^ Department of Cancer Biology, UCL Cancer Institute, University College London, London, United Kingdom; ^3^ Department of Molecular Medicine, Aarhus University Hospital, Aarhus University, Aarhus, Denmark

**Keywords:** epigenetics, DNA methylation, cancer, biomarkers, RNA modifications, chromatine, epidemiology

The epigenome encompasses a compendium of heritable but potentially reversible modifications that collectively regulate gene expression and drive development and cellular differentiation. Human cancers arise due to the accumulation of genetic and epigenetic alterations that ultimately promote malignant transformation (Jones and Baylin, 2002). Epigenetic changes occur in early carcinogenesis with cell-type specific patterns that hold promise to be useful as cancer biomarkers (Berdasco and Esteller, 2019; Zhao et al., 2021). Research into the epigenetic epidemiology of cancer, and the potential application of epigenetic biomarkers in oncology, has been spurred by recent advances in epigenomic methods for the detection of various types of modifications with unprecedented resolution, and the use of alternative tissue sources such as liquid biopsies (Widschwendter et al., 2018).

In this Research Topic, dedicated to “*Epigenetic Biomarkers for Cancer Risk Stratification and Patient Management*”, various authors showcase the recent advances in the development and application of epigenetic biomarkers in cancer management.

An emerging factor in the onset and progression of hepatocellular carcinoma (HCC) are the epigenetic modifications of the RNA molecule. In their study, Qin et al. examined the functions of one hundred RNA modification regulators from eight distinct categories of RNA modifications linked to malignancy in HCC. An expression study showed that almost 90% of RNA regulators had considerably increased expression in tumors compared to normal tissues. Through the process of consensus clustering, they discovered two groups that exhibit clear differences in terms of their biological properties, immunological microenvironment, and prognostic pattern. A scoring system, the RNA modification score (RMScore), was developed to categorize patients into high- and low-risk groups based on their RNA modifications, demonstrating a substantial difference in prognosis between the two groups. They further developed a nomogram that incorporates clinicopathologic characteristics and the RMScore to accurately predict the survival of patients with hepatocellular carcinoma (HCC).

Alterations in tumor DNA methylation (DNAm) patterns are a hallmark of many cancer types. The study by Kim et al. uncovered distinct DNA methylation patterns linked to the prognosis of breast cancer, which are unique to the tumor subtype and menopausal status. The study examined the relationship between the DNA methylation patterns of 692 participants from The Cancer Genome Atlas who had data from the Illumina Infinium HumanMethylation450 BeadChip array employing the Cox proportional hazards model, stratified by tumor subtypes, to analyze all-cause mortality and breast cancer progression. The models were adjusted for several factors, including age, race, stage, menopausal status, tumor purity, and cell type proportion, and evaluated for effect modification through the interaction of DNAm by subtype and menopausal status. They validated the results on a separate dataset (n = 180), discovering a total of fifteen distinct CpG probes that are significantly linked to survival outcomes, and identifying functional pathways that are connected with these DMRs.

Reporting negative findings is essential to maximizing the efficiency of scientific research and preventing duplication of efforts. The study by Webster et al. investigated the potential of DNA methylation from donor whole blood to predict acute graft *versus* host disease (aGVHD) in unrelated donor allogeneic hematopoietic cell transplantation, important for treatment of many malignancies. The study evaluated the DNA methylation over the entire genome in a group of 288 HCT donors with the objective of creating a donor-specific epigenetic classifier in order to decrease the occurrence of aGVHD by enhancing the process of selecting donors. The initially high AUC ROC of 0.91 was not validated in an independent cohort consisting of 288 individuals selected based on the same criteria, where the AUC decreased to 0.51, highlighting the necessity of validating machine learning classifiers independently, especially when creating classifiers for clinical purposes.

Widayati et al. performed an assessment of epigenetic age acceleration in colorectal cancer using open-access data on 1845 samples from 14 studies stored in NCBI GEO and ArrayExpress, and generated a classifier with potential diagnostic capabilities. They computed the epigenetic age (EA) of each sample by employing eleven different epigenetic clock models to determine the corresponding epigenetic age acceleration (EAA) of colorectal cancer (CRC) samples, adjacent normal tissues and normal colon tissues from healthy individuals, reporting stark differences in EAA. A classifier using elastic net regression, incorporating lasso and ridge regularizations was built to predict the diagnosis of CRC by considering the patient’s gender and the EAAs derived from histologically normal controls. This study further underscores the significance of open access clinical data for the advancement of methodology and the education of a new generation of scientists.

We would like to thank all of the authors for providing new findings and intriguing perspectives dedicated to the application of epigenetic biomarkers, which in turn will help to develop innovative management strategies with positive outcomes for oncological patients. We also appreciate the invaluable assistance provided by the independent experts during the peer review of all submitted manuscripts.
